# The effects of postponing BCG vaccination on the risk of BCG-related complications among patients with severe combined immunodeficiency disease in Saudi Arabia

**DOI:** 10.3389/fimmu.2025.1596963

**Published:** 2025-05-09

**Authors:** Amal Aldhaheri, Ohoud Alyabes, Suliman Aljumaah, Raghad Alhuthil, Raghad Alonazi, Shefa Alamoudi, Mohammed Alsuhaibani, Salem Alghamdi, Esam A. Albanyan, Sami Al-Hajjar, Reem Mohammed, Rand Arnaout, Sultan Albuhairi, Nora Alrumayyan, Bandar Al-Saud, Hamoud Al-Mousa

**Affiliations:** ^1^ Department of Pediatrics, Pediatrics Infectious Diseases, Medical College, King Abdulaziz University, Jeddah, Saudi Arabia; ^2^ Section of Infectious Diseases, Department of Pediatrics, King Faisal Specialist Hospital and Research Center, Riyadh, Saudi Arabia; ^3^ College of Medicine, Alfaisal University, Riyadh, Saudi Arabia; ^4^ Section of Pediatric Allergy and Immunology, Department of Pediatrics, King Faisal Specialist Hospital and Research Center, Riyadh, Saudi Arabia

**Keywords:** Bacillus Calmette Guerin, severe combined immunodeficiency disease, vaccination, Saudi Arabia, tuberculosis, inborn errors of immunity

## Abstract

**Introduction:**

The Bacillus Calmette–Guérin (BCG) vaccine is widely used to prevent tuberculosis but is associated with significant complications in patients with severe combined immunodeficiency (SCID). Considering the high incidence of SCID in Saudi Arabia, the Ministry of Health revised its national vaccination schedule in 2019, postponing BCG administration from birth to 6 months of age, aiming to enable time for the diagnosis of primary immunodeficiency diseases before vaccination. This study evaluated the consequences of this policy change on the incidence of BCG-related complications in SCID patients.

**Methods:**

This retrospective study included 178 SCID patients diagnosed at King Faisal Specialist Hospital and Research Center, Riyadh, between 2015 and 2023. Patients were divided into two cohorts: Era 1 (2015–2019), when BCG vaccination was administered at birth, and Era 2 (2019–2023), when BCG vaccination was administered at 6 months of age. Data on demographics, clinical presentations, BCG-related complications, genetic testing, treatment, and outcomes were analyzed.

**Results:**

A total of 49 SCID patients developed BCGitis, of which 65.3% experienced disseminated disease. The incidence of BCG-related complications dropped significantly after the policy change, from 46.1% in Era 1 to 2.6% in Era 2. Patients required stem cell transplantation and a median of 17.6 months of anti-mycobacterial therapy. The crude mortality rate was high (36.7%; 18/49), with 66.7% (12/18) of these fatalities linked to disseminated BCGitis.

**Conclusions:**

Postponing BCG vaccination to 6 months of age significantly decreases the incidence of BCG-related complications in SCID patients and highlights the importance of tailoring vaccination schedules for high-risk populations. Early newborn screening and timely diagnosis of immunodeficiencies are essential to further minimize complications. The revised vaccination policy of Saudi Arabia provides a model for optimizing immunization strategies in regions with a high prevalence of inborn errors of immunity.

## 1 Introduction

Tuberculosis (TB) is a global health issue, and Saudi Arabia is no exception, with a reported incidence of 14 cases per 100,000 people (1). Since 1912, the only available vaccine for TB has been the Bacillus Calmette Guerin (BCG) vaccine, which contains live attenuated *Mycobacterium bovis* (2, 3). The BCG vaccine has been used to combat TB and is considered one of the most extensively used vaccinations in the world today. Since the 1960s, it has been routinely administered in most nations, mostly to newborns through national childhood vaccination programs. BCG vaccination protects against meningitis and disseminated TB but does not prevent primary infection or, more crucially, the reactivation of latent pulmonary infection, which is the major source of community transmission. The BCG vaccine was first introduced to Saudi Arabia in 1964 and was initially designated to high-risk populations but was then expanded to all newborns in 1970 (4). However, the vaccine has been associated with significant adverse effects, including local ulceration at the vaccine site, regional lymphadenitis, osteomyelitis, and disseminated disease with an overall rate of 0.5–100 cases per 1,000 vaccinations (5). In Saudi Arabia, the BCG vaccine is part of the nationwide vaccination schedule and is administered to all infants at birth. An increased prevalence of BCG-related lymphadenitis (0–10.4 cases per 1,000 vaccinations) has been observed since the Danish strain (SSI 1331) started to be used in November 2005 (4).

Severe combined immunodeficiency (SCID) is a group of hereditary diseases defined by inhibited T lymphocyte differentiation, which is sometimes coupled with abnormal development of other lymphocyte lineages, such as B or natural killer (NK) cells (6, 7). Early-onset infections, interstitial pneumonitis, oral candidiasis, and persistent diarrhea with growth retardation are all common clinical presentations. SCID is characterized by a high level of genetic and clinical heterogeneity, as more than 14 genetic defects have been identified and can be distinguished according to cellular phenotype, inheritance pattern, and the responsible genes (8, 9). Live attenuated vaccinations are strictly contraindicated in SCID patients. However, because the BCG vaccine is often administered at a very young age, SCID patients are immunized before being diagnosed with SCID. SCID patients have a very high incidence of complications due to BCG vaccination, exhibiting higher rates of morbidity and mortality. In a comprehensive study investigating the effects of BCG in 349 SCID patients who were vaccinated prior to diagnosis, 51% experienced complications, of which 34% were disseminated, and 17% were localized. T cell counts of <250/µL and BCG vaccination within the first month of life were identified as risk factors for BCG disease development. Among the 160 patients treated with anti-mycobacterial treatment for symptomatic BCG infection, 46 BCG-associated fatalities were documented (10).

Consanguineous marriages are prevalent in Saudi Arabia, with a total rate of 60% (11). Most monogenic SCID causes are autosomal recessive and are thus likely to be more frequent in regions with high rates of consanguineous marriages (12). Our previous pilot T cell receptor excision circle-based newborn screening project revealed a high incidence of SCID among the Saudi population (1/2,906 live births), which is 20-fold higher than the incidence reported in the USA (13, 14). For this reason, in August 2019, the Ministry of Health in Saudi Arabia postponed BCG vaccination to 6 months of age, thereby enabling more time for potential diagnoses of primary immunodeficiency diseases. However, limited studies have evaluated the effects of administering the BCG vaccine at birth compared to later ages (15, 16). The aim of this study was to assess the effects of delaying BCG vaccination to the age of 6 months on the risk of developing BCG-related complications among SCID patients.

## 2 Methods

### 2.1 Study design and population

**Figure 1:**
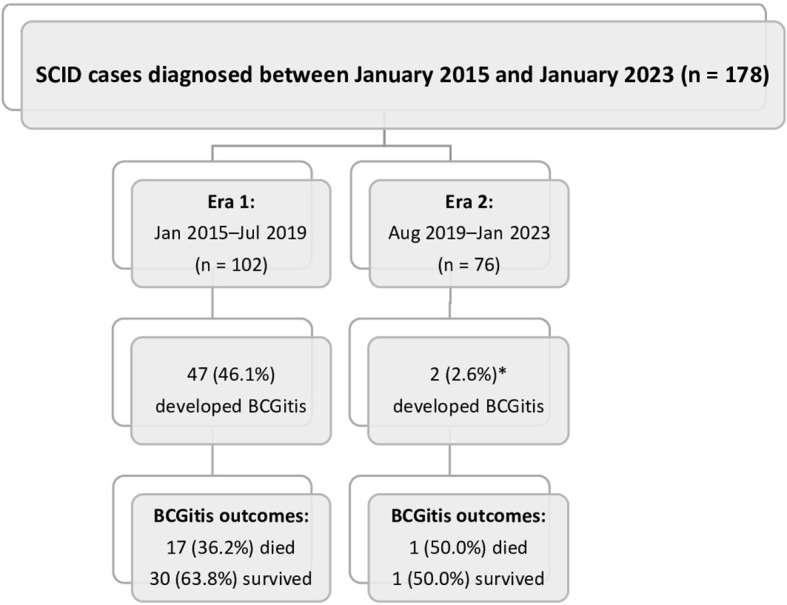
Summary of the study cohort. *A significant decrease in the incidence of Bacillus Calmette–Guérin infections (BCGitis) was noted in Era 2 (2.6%) compared with Era 1 (46.1%; *p* < 0.001).

This was a retrospective study that reviewed all SCID patients who were diagnosed at the King Faisal Specialist Hospital and Research Center (KFSH-RC) in Riyadh, Saudi Arabia, over 8 years (from January 2015 to January 2023). The study compared the incidence of BCG-related complications in SCID patients between two periods (1): Era 1, from January 2015 to July 2019 (prior to the schedule change), during which all newborns received BCG vaccination at birth, and (2) Era 2, from August 2019 to January 2023 (after the updated national vaccination schedule), during which BCG vaccination was administered to all infants at the age of 6 months (**Figure 1**). This study included all SCID patients who had documented BCG-related complications following BCG vaccination and excluded patients who did not receive the BCG vaccine. This study was approved by the Institutional Review Board at KFSH-RC (reference number: 2231217, approval date: July 12, 2023).

### 2.2 Definitions

SCID was defined according to the diagnostic criteria of the Primary Immune Deficiency Treatment Consortium (17). BCG-related disease was defined as local, regional, or disseminated using the pediatric criteria developed by Hesseling and colleagues (18). Local BCG disease involves a BCG injection site abscess of > 10 mm or severe BCG scar ulceration. Regional disease involves the enlargement, suppuration, or fistula formation of ipsilateral regional lymph node(s). Disseminated disease involves distant anatomical site(s) beyond the BCG injection site and ipsilateral lymph node(s).

### 2.3 Data collection and analysis

Data were collected through electronic chart reviews and entered into the REDCap database (10.8.0, ^©^ 2021 Vanderbilt University). The extracted data included demographics, medical history, immunological and genetic testing, BCG vaccine-related complications, treatment, antimicrobial susceptibility, stem cell transplantation, and outcomes. Stata 18 (^©^ 2025 StataCorp) was used for analyses. Categorical variables were reported as frequency and percentage (%), and continuous variables were reported as medians and interquartile ranges (IQR). Graphs were generated using Microsoft Excel 2016. Univariable analysis was performed using Fisher’s exact test. A *p*-value of <0.05 was considered significant.

## 3 Results

Between January 2015 and January 2023, 179 SCID patients were diagnosed at KFSH-RC. Among the 102 patients in Era 1, 47 (46.1%) suffered from BCG-related disease. Among the 76 patients in Era 2, only 2 (2.6%) suffered from BCG-related complications (p < 0.001) (**Figure 1**).

Of the 49 BCGitis cases, 22 (44.9%) were boys, and 27 (55.1%) were girls, with a median age of BCG-related clinical presentation of 5 months (IQR: 4–7). The overall median time from BCG vaccination to the development of disease manifestations was 5 months (IQR: 4–6) (**Table 1**). BCG-related complications in SCID patients varied in severity: 34.7% exhibited localized or regional disease, whereas 65.3% exhibited disseminated disease (**Table 2**). SCID (T-, B-, NK+) was the most prevalent phenotype, with *RAG1* and *DCLRE1C* being the most common genetic defects (**Table 1**). The median (interquartile range) for CD3, CD4 and CD8 counts were 116 (17, 1064), 66 (8, 577) and 11 (0, 149)/mm^3^ respectively. Diagnoses were made mainly through culture (61.2%), gastric aspirate (8.2%), or clinical diagnosis (30.6%) (**Table 2**).

**Table 1:** Characteristics of BCGitis cases (n = 49).

Characteristics	n (%), Median [IQR]
Gender	
Male	22 (44.9)
Female	27 (55.1)
Age at BCG vaccine	
At birth	47 (95.9)
At 6 months	2 (4.1)
Age at SCID diagnosis (months)	5 [4, 7]
BCGitis presentation age (months)	5 [4, 6]
SCID Type	
T−, B−, NK+	23 (46.9)
T−, B+, NK+	3 (6.1)
T−, B+, NK−	6 (12.2)
T−, B−, NK−	3 (6.1)
T+, B−, NK+ (Omenn)	11 (22.5)
T+, B−, NK− (Omenn)	1 (2.0)
T+,B+,NK+ (Omenn)	1 (2.0)
CD8 deficiency	1 (2.0)
SCID Gene Mentioned (n = 40)	
*ADA* deficiency	3 (7.5)
*CD3E*	1 (2.5)
*DCLRE1C*	6 (15)
*DNA* ligase 1 deficiency	1 (2.5)
*IL2RG* chain mutation	2 (5.0)
*JAK3*	5 (12.5)
*LAT*	1 (2.5)
*NHEJ1*	1 (2.5)
*PNP*	2 (5.0)
*RAG1*	15 (37.5)
*RAG2*	2 (5.0)
*ZAP-70* deficiencies	1 (2.5)

IQR, interquartile range; BCGitis, Bacille Calmette–Guérin infection; BCG, Bacille Calmette–Guérin vaccine; SCID, severe combined immunodeficiency; NK, natural killer cells; *ADA*, adenosine deaminase; *CD3E*, CD3 epsilon subunit; *DCLRE1C*, DNA cross-link repair 1C; *IL2RG*, interleukin-2 receptor subunit gamma; *JAK3*, Janus kinase 3; *LAT*, linker for activation of T cells; *NHEJ1*, non-homologous end-joining factor 1; *PNP*, purine nucleoside phosphorylase; *RAG1*, recombination activating gene 1; *RAG2*, recombination activating gene 2; *ZAP-70*, zeta-associated protein 70.

**Table 2:** Management and outcomes of BCGitis (n = 49).

Investigations	n (%), Median [IQR]
BCGitis diagnosis	
Culture	30 (61.2)
AFP gastric Aspiration	4 (8.2)
Clinical	15 (30.6)
HSCT	44 (89.8)
Type of HSCT (n = 44)	
Full Matched	26 (59.1)
Haploidentical	12 (27.3)
Umbilical Cord Transplant	6 (13.6)
Target treatment	
Clarithromycin	46 (93.9)
Ethambutol	38 (77.6)
Rifampicin	30 (61.2)
Ciprofloxacin	22 (44.9)
Levofloxacin	20 (40.8)
Isoniazid	19 (38.8)
Amikacin	13 (26.5)
Cycloserine	10 (20.4)
Moxifloxacin	6 (12.2)
Linezolid	2 (4.1)
Ceftazidime-Avibactium	1 (2.0)
Treatment status	
Completed	30 (61.2)
Died during treatment	17 (34.7)
Stopped by the family	1 (2.0)
Ongoing	1 (2.0)
Treatment duration (months) (n=30)	17.6 [13.5, 24.2]
Regional/Local disease	17 (34.7)
Regional disease	16 (94.1)
Local BCG disease	1 (5.9)
Disseminated disease	32 (65.3)
Dissemination site (n = 32)*	
Liver/spleen	8 (25.0)
Bone	8 (25.0)
Lymph nodes	12 (37.5)
Skin	10 (31.3)
Muscles	1 (3.1)
Lung(s)	10 (31.3)
Intrabdominal collection	1 (3.1)
Crude mortality	18/49 (36.7)
Contributing causes of mortality Invasive fungal intracranial infection Lack of full match donor Pneumonia and septic shock Post HSCT veno-occlusive disease Measles	1 2 10 1 1

*Twelve patients had more than one dissemination site.

HSCT, hematopoietic stem cell transplantation; BCG, Bacillus Calmette–Guérin; AFP, acid-fast bacilli.

Treatment included hematopoietic stem cell transplantation and anti-mycobacterial therapy. Hematopoietic stem cell transplantation was performed in 89.8% of patients. The most commonly used anti-TB treatments were clarithromycin (93.9%), ethambutol (77.6%), and rifampicin (61.2%). Treatment durations were prolonged, with a median of 17.6 months (IQR: 13.5–24.2) (**Table 2**). All culture isolates demonstrated 100% sensitivity to cycloserine, clarithromycin, ciprofloxacin, moxifloxacin, amikacin, ethambutol, ethionamide, and streptomycin. Additionally, 96.0% and 85.2% of the isolates were sensitive to rifampicin and isoniazid, respectively. However, all the isolates were resistant to pyrazinamide. The most commonly used combination of anti-mycobacterial therapy for localized or regional BCG disease was ethambutol, clarithromycin, and ciprofloxacin, administered in 62.5% of patients. In cases of disseminated disease, a four-drug regimen comprising rifampicin, ethambutol, ciprofloxacin, and clarithromycin was employed in 65.6% of patients.

Among SCID patients with BCG complications, the most prevalent subtype was SCID (T-, B-, NK+), accounting for 50.0% of those with disseminated disease and 41.2% of those with localized or regional disease. This difference was not statistically significant (p = 0.764). However, the SCID subtype T-, B+, NK- was more frequently associated with disseminated disease (18.8%) than localized or regional disease (0.0%), showing a trend toward significance (p = 0.080) (**Table 3**). The crude mortality rate was 36.7% (18/49). Among these fatalities, 66.7% (12/18) were attributed to disseminated BCGitis (**Table 2**).

**Table 3:** Relationship between types of severe combined immunodeficiency (SCID) and disseminated Bacillus Calmette–Guérin infections (BCGitis) (n = 49).

SCID Type	Disseminated Bcgitis (n = 32)	Regional/local changes (n = 17)	*p*-value
T−, B−, NK+	16 (50.0)	7 (41.2)	0.764
T−, B+, NK+	2 (6.3)	1 (5.9)	1.000
T−, B+, NK−	6 (18.8)	0 (0.0)	0.080
T−, B−, NK−	2 (6.3)	1 (5.9)	1.000
T+, B−, NK+ (Omenn)	6 (18.8)	5 (29.4)	0.480
T+, B−, NK− (Omenn)	0 (0.0)	1 (5.9)	0.347
T+,B+,NK+ (Omenn)	0 (0.0)	1 (5.9)	0.347
CD8 deficiency	0 (0.0)	1 (5.9)	0.347

## 4 Discussion

The prevalence of BCG complications in the general population varies significantly depending on the nation and vaccine strain used. However, the rates of localized and disseminated complications are estimated at 1 in 2,500 and 1 in 100,000 vaccinations, respectively (19). All SCID patients with varying underlying genetic abnormalities have a defective adaptive immune response, which impairs mycobacterial infection control. As a result, the frequency of BCG-related complications in SCID patients is greater than that in the general population (10, 15, 20–23). The combined experience of 28 centers in 17 countries revealed that one out of every two BCG-vaccinated SCID patients develops BCG-related manifestations, with two-thirds developing disseminated complications (representing a 33,000-fold higher risk compared to the general population) and the other one-third developing localized complications (representing a 400-fold higher risk compared to the general population). Two factors are strongly associated with the higher occurrence of BCG complications: the total number of T cells at the time of SCID diagnosis and age at the time of BCG vaccination. SCID patients who were vaccinated within the first month of life had a significantly greater risk of BCG-related complications, which was associated with an increased likelihood of vaccine-related mortality (10).

Consanguineous marriages are a strongly ingrained custom in Middle Eastern and North African nations, contributing to an increase in the occurrence of autosomal recessive disorders such as inborn errors of immunity (12). A newborn screening pilot study in Saudi Arabia revealed a high prevalence of SCID (1/2,906 live births) (13). We previously reported that disseminated *M. bovis* was found in 49 out of 114 (43%) SCID patients receiving the BCG vaccine at birth at KFSH-RC between 2010 and 2013 (24). In Saudi Arabia, the BCG vaccine has traditionally been administered to newborns at birth. However, recent guidelines from the Saudi Ministry of Health recommend postponing BCG vaccination until the age of 6 months, aiming to enhance vaccine safety and efficacy. This policy shift in Saudi Arabia has significantly decreased vaccine-related complications, particularly in SCID patients. Our study demonstrates a significant decrease in the incidence of BCGitis, from 46.1% before the policy change to 2.6% post-implementation. This suggests a strong association between the timing of BCG vaccination and adverse outcomes in vulnerable populations. Such findings emphasize the importance of tailoring immunization schedules to align with the safety and needs of high-risk groups, particularly for countries with a high incidence of inborn errors of immunity. Our study also underscores the importance of continuously evaluating vaccination policies to ensure optimal protection while minimizing risks.

Delaying BCG vaccination to the age of 6 months can have both potential benefits and risks in terms of TB prevention in the general population. Delaying vaccination may leave infants unprotected during the critical first months of life, especially in regions with high TB incidence. In regions with low TB incidence, delaying BCG vaccination may have negligible effects on overall TB rates (15). Conversely, in regions with widespread TB, delaying vaccination could lead to an increase in TB cases among unvaccinated infants, affecting community-level TB control efforts. However, studies suggest that administering the BCG vaccine at an older age may lead to a more robust immune response, potentially improving its efficacy in preventing TB. This could be due to the enhanced maturity of an infant’s immune system at 6 months compared to the neonatal period (16). Delaying BCG vaccination to 6 months of age in countries with a high incidence of inborn errors of immunity can significantly decrease vaccine-related complications but must be carefully weighed against the risk of TB exposure in infancy. In such settings, tailoring vaccination policies to the local epidemiology of TB and incidence of inborn errors of immunity is critical for minimizing risks while ensuring effective TB prevention.

Previous studies have shown that the risk of BCG-related complications is independent of the SCID subtype (10, 23). In our cohort, the T-, B+, NK- SCID phenotype showed a borderline association with disseminated disease (p = 0.080). This might be related to a lack of NK cells, which act at the interface of innate and adaptive immunity to control infections and shape immune responses and hence play a significant role in controlling mycobacterial infections (25, 26). In our study, only two patients (2.6%) developed BCG-related complications after postponed vaccination; both cases were diagnosed with SCID relatively late, i.e., at 10 and 22 months of age. These patients presented with BCGitis at 12 and 22 months, respectively. The first patient had *JAK3* SCID and developed regional BCGitis in the ipsilateral lymph nodes, whereas the second patient had *PNP* SCID and developed disseminated BCGitis, which resulted in death. These two cases underscore the critical importance of early immunodeficiency screening to optimize vaccination timing and minimize complications.

The management of SCID patients and BCG-related complications includes providing immune reconstitution through hematopoietic stem cell transplantation, gene therapy, or enzyme replacement in addition to anti-TB therapy. The antimicrobial therapy used to manage BCG-related complications in patients with inborn errors of immunity (IEI) remains unclear and controversial. This uncertainty stems from the heterogeneity of underlying immune defects, variability in clinical presentation and limited high-quality evidence guiding treatment decisions. In response, several expert guidelines and position papers have been published to provide consensus-based recommendations for the diagnosis and management of these complications (27–29). We followed the European Society of Immunodeficiency guidelines, which base the recommended regimen intensity on disease severity: two antimicrobials for localized skin manifestations, three for ipsilateral lymphadenitis, and four for disseminated BCGitis. The treatment durations in the cohort of this study varied greatly: 4.3–17.1 months for localized and regional BCGitis and 11.5–38.3 months for disseminated cases. These durations were influenced by clinical responses, immunological reconstitutions, adverse effects, and therapeutic interactions. The antimicrobial susceptibility studies showed that the BCG strains were 100% sensitive to cycloserine, clarithromycin, ciprofloxacin, moxifloxacin, amikacin, ethambutol, ethionamide, and streptomycin. However, the isoniazid and rifampicin resistance rates were 14.8% and 4.0%, respectively. Isoniazid was underused in our cohort due to concerns regarding the potential development of resistance, as well as the risk of hepatotoxicity and other adverse effects. The universal resistance to pyrazinamide aligns with the known intrinsic resistance of BCG strains. This suggests potential variability in resistance patterns and reinforces the importance of local susceptibility testing to guide treatment choices.

The crude mortality rate was notably high (36.7%; 18/49), with 66.7% (12/18) of these fatalities linked to disseminated BCGitis. Similarly, Cocchi et al. reported a mortality rate of 27.8% (10 deaths in 36 patients) (23). Conversely, Botaro et al. studied BCG vaccination results in 11 SCID infants in Brazil and reported a higher mortality rate (73%), with 25% of deaths related to BCG vaccination (21). This significant fatality rate highlights the importance of proactive screening and early management to reduce the risks associated with BCG vaccination in SCID patients. Thus, diagnosing systemic infections caused by BCG vaccination in SCID patients demands a high degree of clinical vigilance and appropriate diagnostic tools, such as mycobacterial cultures, biochemical identification, or polymerase chain reaction. These diagnostic procedures should be initiated promptly in cases of disseminated BCG disease, alongside timely treatment with anti-TB medications and, when feasible, therapies that address the underlying immunodeficiency (14).

BCG-related complications are not limited to patients with severe combined immunodeficiency (SCID) but have also been reported in a broader spectrum of inborn errors of immunity (IEI), including combined immunodeficiencies (CID) and phagocytic defects such as chronic granulomatous disease (CGD) and Mendelian susceptibility to mycobacterial disease (MSMD) (29–33). These conditions, while sometimes diagnosed later in infancy or childhood due to more subtle clinical presentations, still predispose patients to disseminated or severe localized BCG disease if vaccinated early. While delaying BCG vaccination to six months of age—as implemented in our study—appears to reduce complications in SCID, it may not be sufficient to protect patients with other susceptible IEIs who remain undiagnosed beyond that age. Therefore, similar vaccine-delaying strategies should be investigated in other high-risk IEI populations, particularly in regions with high consanguinity and IEI prevalence. In such settings, a broader discussion is warranted around whether routine BCG vaccination should be postponed even further or potentially withheld altogether until immune competence is confirmed, to mitigate the risk of severe vaccine-related adverse events.

### 4.1 Limitations

This study has provided insights into the beneficial effects of postponing BCG vaccination on the risk of BCG-related complications among SCID patients in Saudi Arabia. However, the consequences of postponing BCG vaccination on the risk of TB in the general population must be investigated in future studies.

## 5 Conclusions

This study demonstrates that postponing BCG vaccination to 6 months of age significantly decreases the incidence of BCGitis in SCID patients, highlighting the importance of adjusting vaccination schedules for high-risk populations. In Saudi Arabia, the policy change to administer the BCG vaccine at 6 months of age allows time for the early diagnosis of immunodeficiencies in infants and may serve as a model for global vaccination strategies in similar contexts. Further research is needed to refine vaccination timing, develop risk stratification methods, and explore alternative immunization approaches to minimize complications in immunocompromised individuals. Additionally, long-term monitoring and earlier screening for immunodeficiencies remain critical for preventing severe vaccine-related complications in this vulnerable group.

## Data Availability

The data analyzed in this study is subject to the following licenses/restrictions: Data can be available from the corresponding author upon a reasonable request. Requests to access these datasets should be directed to hamoudalmousa@kfshrc.edu.sa.
